# Plasma levels of soluble Flt-1 and Tie-2 correlate with neovascularization in human carotid atherosclerotic plaques – a pilot study

**DOI:** 10.3389/fimmu.2026.1791571

**Published:** 2026-07-21

**Authors:** Mahtab Zamani, Karolina Skagen, Beate Lindberg, Vigdis Bjerkeli, Tuva B. Dahl, Annika Elisabeth Michelsen, Thor Ueland, Pål Aukrust, Bente Halvorsen, Mona Skjelland

**Affiliations:** 1 Department of Neurology, Oslo University Hospital, Oslo, Norway; 2 Institute of Clinical Medicine, University of Oslo, Oslo, Norway; 3 Department of Cardiothoracic Surgery, Oslo University Hospital, Oslo, Norway; 4 Research Institute of Internal Medicine, Oslo University Hospital, Oslo, Norway; 5 Thrombosis Research Center, Division of Internal Medicine, University Hospital of North Norway, Tromsø, Norway; 6 Section of Clinical Immunology and Infectious Diseases, Oslo University Hospital, Oslo, Norway

**Keywords:** carotid atherosclerosis, IPN, neovascularization, sFlt-1, SMI, sTie-2, SMI (superb microvascular imaging)

## Abstract

**Objective:**

To determine whether circulating soluble Flt−1 (sFlt−1) and soluble Tie−2 (sTie−2) are associated with intraplaque neovascularization (IPN) in human carotid atherosclerotic plaques.

**Methods:**

Forty−four patients with ≥50% carotid stenosis underwent conventional carotid ultrasound and superb microvascular imaging (SMI); 29 had plasma collected and 11 provided carotid endarterectomy specimens for histology. IPN was quantified as neovessel counts in 2−minute SMI cine loops and as microvessel counts in excised plaques. Plasma VEGF−A/B/C/D, Ang−2, sFlt−1, and sTie−2 were measured by immunoassays. Public single−cell RNA−seq data from human carotid plaques were interrogated to map FLT1, TEK, VEGFA, ANGPT1, and ANGPT2 expression to specific plaque cell populations.

**Results:**

IPN was detected in 33/44 (75%) patients, with 0–16 neovessels on SMI (median 4). Plasma sFlt−1 correlated with SMI neovessel counts (r=0.42, p=0.030), with a similar trend for sTie−2 (r=0.37, p=0.057), whereas VEGF−A/B/C/D and Ang−2 showed no significant relationships. In the surgical subgroup, histological neovessel counts correlated with sFlt−1 (r=0.65, p=0.030) and SMI neovessel count (*r= 0.68, p =0.02)* but not sTie−2. sFlt−1 and sTie−2 correlated with BMI (and sTie−2 also with age), yet log−sFlt−1 remained independently associated with SMI−derived neovessel counts after adjustment for age and BMI (B = 13.24, 95% CI 0.17–26.30, p=0.047; R²=0.18), while sTie−2 was not. No significant associations were observed between IPN or sFlt−1/sTie−2 and lipid profile, CRP, leukocyte counts, or other conventional risk factors. Single−cell transcriptomics showed FLT1 and ANGPT2 broadly expressed across endothelial, smooth−muscle, and myeloid clusters, with TEK largely confined to endothelial cells and ANGPT1 to smooth−muscle cells, supporting local activation of VEGF–FLT1 and Ang–TEK/Tie−2 signaling within plaques.

**Conclusion:**

In this pilot study, higher plasma sFlt−1, and, to a lesser degree sTie−2, correlated with carotid IPN on SMI and histology, independent of age and BMI. These findings suggest that soluble VEGF− and Ang/Tie−2–receptor pathways may serve as circulating, context−sensitive markers of intraplaque angiogenesis and plaque vulnerability, meriting evaluation in larger longitudinal cohorts.

## Introduction

Atherosclerosis remains a principal cause of morbidity and mortality worldwide, characterized by chronic inflammatory responses and vascular remodeling, leading to the development of unstable atherosclerotic plaques (1–3). These plaques not only compromise arterial integrity but also predispose individuals to ischemic events through luminal obstruction and acute plaque rupture. In fact, 10-20% of ischemic strokes are due to thromboembolism from an unstable carotid plaque (4, 5).

A critical but often underexplored facet of atherosclerotic plaque progression is intraplaque neovascularization (IPN) - the pathological formation of microvascular networks originating from the vasa vasorum into the necrotic core. Although neovascularization may initially arise as a compensatory response to the hypoxia within the growing plaque (6), it paradoxically contributes to plaque destabilization (7). These newly formed neovessels been described in histopathological studies as structurally immature and fragile, making them prone to rupture and intraplaque hemorrhage (IPH), which exacerbates inflammation, increases plaque volume, and heightens the risk of thromboembolic events (8, 9). Detecting these micro-vessels with small blood flow signals using standard Doppler ultrasound methods is challenging. In our recent study, we introduced a novel ultrasound method, Superb Microvascular Imaging (SMI), which utilizes an algorithm that effectively overcomes the challenges faced by standard ultrasound in the visualization and quantification of IPN. We demonstrated that SMI is comparable to contrast-enhanced ultrasound for the assessment of IPN (10).

At the molecular level, regulation of angiogenesis within atherosclerotic plaques is mediated by a complex interplay of growth factors and their receptors. Among key regulators of angiogenesis are Vascular Endothelial Growth Factor (VEGF) and Angiopoietins (Ang-1 and Ang-2), whose effects are mediated through specific tyrosine kinase receptors. VEGF primarily signals through VEGFR-1, fms-like tyrosine kinase-1 (Flt-1) receptors and VEGFR-2/KDR (kinase insert domain receptor), while Angiopoietins act via endothelial-specific receptor tyrosine kinase (Tie-1 and Tie-2) receptors (11, 12). The balance between Ang-1 and Ang-2, along with Tie-2 activity, plays a critical role in determining whether angiogenesis results in stable, functional vasculature or in the formation of leaky, abnormal vessels. Soluble Flt-1 (sFlt-1) and soluble Tie-2 (sTie-2) are circulating soluble form of the receptor tyrosine kinase, which typically exists as a membrane-bound receptor on endothelial cells. sFlt-1, a naturally occurring decoy receptor for VEGF, acts as an anti-angiogenic factor by sequestering VEGF, preventing binding to its receptor VEGFR-2 on endothelial cells (13). Similarly, sTie- -2 binds to Ang-1 and Ang-2, blocking their interaction with the membrane-bound Tie-2 receptor and subsequently inhibiting angiogenesis (14). In a population-based cohort (Dallas heart study), elevated sFlt-1 levels were associated with subclinical aortic atherosclerosis and increased risk of future ischemic events (15). sTie-2 has been implicated in the pathogenesis of cardiovascular disease (16). However, the specific role of circulating plasma levels of sFlt-1, sTie-2 receptors in the concept of intraplaque neovascularization remains poorly understood and needs to be elucidated.

Building on these foundational insights, we hypothesized that a dysregulated angiogenesis, as assessed by systemic levels of key angiogenic modulators, specifically sFlt-1 and sTie-2, is involved in intraplaque neovascularization, as assessed quantitatively by SMI. This hypothesis was examined in a well-characterized cohort of patients with clinically significant carotid atherosclerosis with and without abnormal intraplaque neovascularization.

## Methods

### Study population

We included 44 patients attending the Department of Neurology, Oslo University Hospital before carotid endarterectomy (CEA) or at a routine outpatient control for the asymptomatic patients. Between January 2016 and January 2019, 31 patients with >50% internal carotid artery stenosis were prospectively included. All patients underwent conventional Doppler ultrasound, contrast-enhanced ultrasound (CEUS), Superb Microvascular Imaging (SMI), and routine blood testing. Twenty-two patients underwent carotid endarterectomy, of whom plaque tissue was available for histological analysis in 20 cases (Published in 2019) (10). Additional blood samples for biomarker analyses were obtained in 16 of the 31 patients.

Between January and April 2019, 13 additional patients were included. These patients underwent conventional Doppler ultrasound, SMI, routine blood testing, and biomarker sampling.

**Figure 1:**
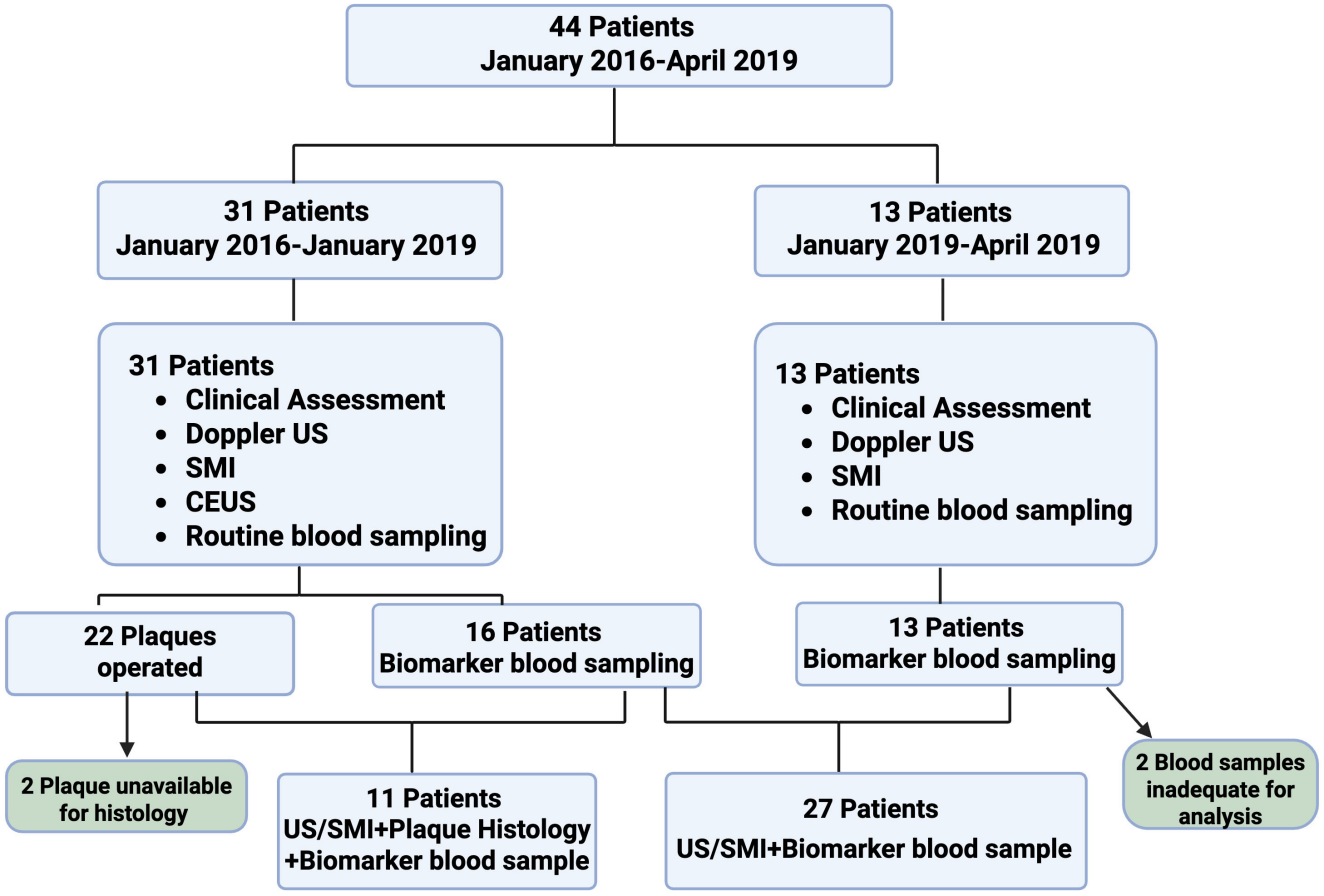
Flowchart.

Overall, 29 patients had both biomarker samples and SMI assessment of intraplaque neovascularization (IPN). Of these, 11 patients had available plaque histology (all from the initial cohort). Two biomarker samples were excluded due to insufficient volume, resulting in 27 patients available for analysis of sFlt-1, Tie-2, VEGF-A, VEGF-B, VEGF-C, VEGF-D, and angiopoietin-2. Thirty patients of the total cohort were symptomatic, i.e had undergone ipsilateral cerebral ischemia (ischemic strokes, transitory ischemic attacks, or amaurosis fugax) within 3 months prior to study inclusion and 14 patients were asymptomatic. (**Figure 1**: flowchart).

The study protocol conforms to the ethical guidelines of the Declaration of Helsinki. The study was approved by the Norwegian Regional Committee for Medical and Health Research Ethics (ID REC 2014/1468), and written informed consent was obtained from all patients. This study is funded by South-Eastern Norway Regional Health Authority (ID 2010011).

### Ultrasonographic investigation, including SMI

Ultrasonography Imaging was performed with a Canon ultrasound system Aplio 500 (Canon Medical Systems, Otawara, Japan) using a 7.5 MHz linear probe on both carotid arteries for standard Doppler ultrasound and SMI ultrasound. The common carotid artery, carotid bifurcation, and internal carotid arteries were examined in longitudinal and transverse planes in standard ultrasound. The degree of carotid artery stenosis was determined based on peak-systolic and end-diastolic velocities according to consensus criteria of the Society of Radiologists in ultrasound (17).

**Figure 2:**
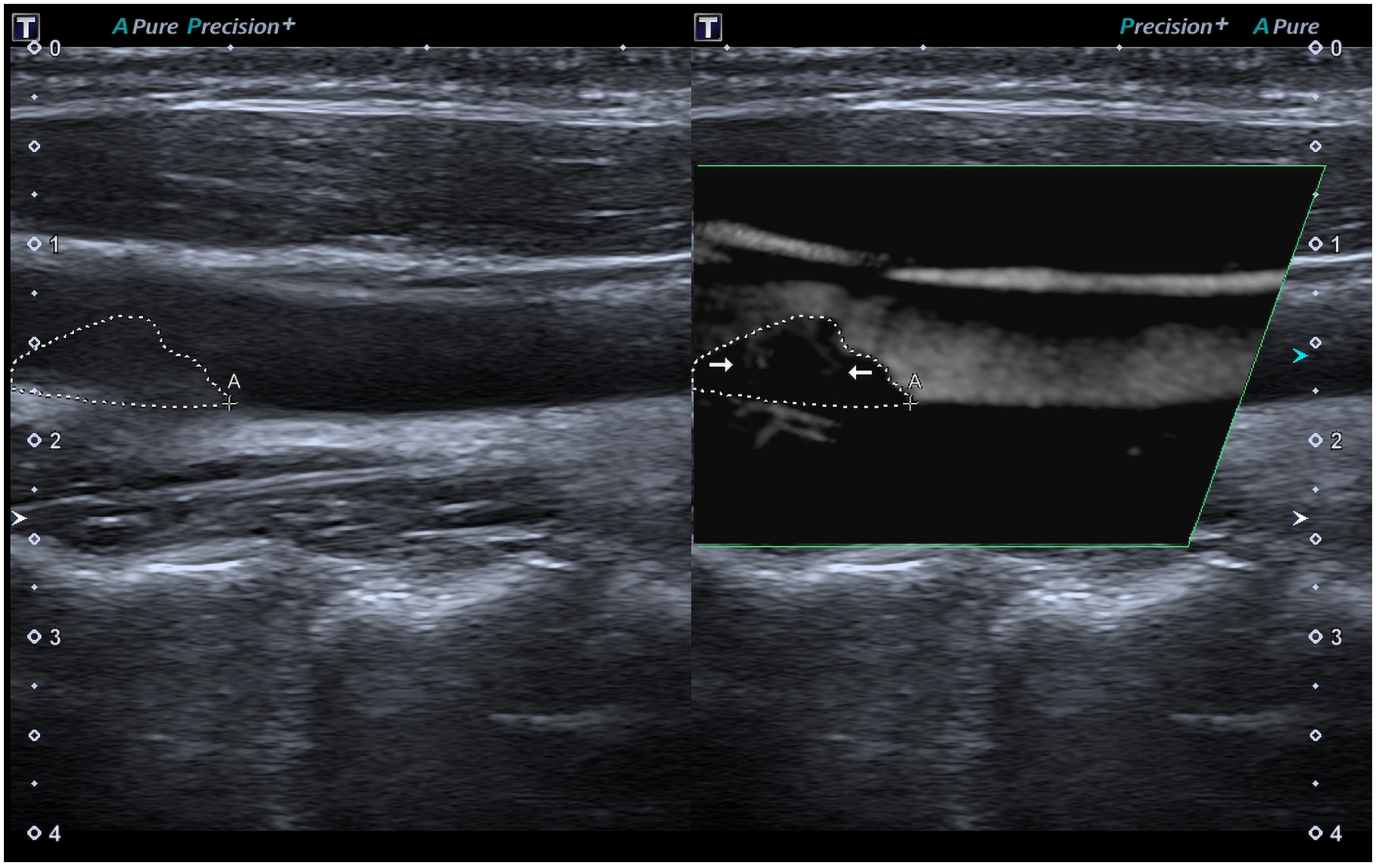
Representative SMI image of intraplaque neovascularization. B-mode ultrasound image (left) and corresponding SMI image (right) of a carotid plaque. The plaque is outlined by the dotted line, and arrows indicates microvascular signal consistent with intraplaque neovascularization (IPN).

SMI was performed in monochrome mode with twin−view B−mode/SMI display. After optimizing SMI settings (mechanical index 1.5, frame rate 50–60 fps, dynamic range 55–65 dB, SMI velocity 0.8–2.0 cm/s), plaques were observed in transverse and longitudinal planes for 2 minutes and cine loops were stored. Moving intraplaque microvessel flow (IMVF) signals were visually counted over the 2−minute recording (**Figure 2**). This protocol is identical to that used in our previous SMI validation study, where two blinded readers demonstrated good inter− and intra−observer reproducibility for both SMI−derived IMVF counts and histological neovessel quantification (10).

### Blood sampling protocol and biochemical analyses

EDTA plasma was collected and kept on melting ice, centrifuged within 30 minutes at 2000*g* for 20 minutes to obtain platelet-poor plasma and stored at -80 °C until analysis. All samples were thawed <three times and assessed in duplicate. VEGF-A, VEGF-B, VEGFF-C, VEGF-D, Ang-2, sFlt-1, sTie-2, were analyzed by U-Plex Metabolic Group 1 (human) assay (Meso Scale Diagnostics, Rockville, MD). VEGF-D and Ang-2 were assessed by enzyme immunoassays with matched antibody pairs from RnD systems (Stillwater, MA).

Blood samples were also analyzed by routine methods at the Department of Clinical Biochemistry, Oslo University Hospital. These included Low-Density Lipoprotein (LDL) cholesterol, High-Density Lipoprotein (HDL) cholesterol, Triglycerides (TG), Total cholesterol, fasting glucose, glycosylated Hemoglobin (HbA1c), estimated Glomerular filtration rate (eGFR), high-sensitivity C-reactive protein (CRP), leukocyte and platelet counts, sedimentation rate (ESR) and electrolytes.

### Assessment of RNA expression across cell subsets in carotid plaque by publicly available single cell transcriptome data

Flt-1, TEK (TEK receptor tyrosine kinase), VEGF-A, Ang-1 and Ang-2 RNA expression in cell subsets of carotid plaque was identified using the publicly available single cell RNAseq data of atherosclerotic plaque from 38 patients undergoing Carotid Artery Plaque Endarterectomy (PMID: 35174364) and the PlaqueView platform (PMID: 34871816) with Seurat_with_Tabular_Ref_Clusters labeling and human primary Cell Atlas for cell identification (18, 19).

### Tissue processing and histological analysis

Carotid plaques removed en bloc (intact) at endarterectomy were assessed for inflammation and granulation tissue as described previously (20). Neovessels were examined at 100× magnification. For each plaque, neovessels were identified using morphological criteria on hematoxylin and eosin–stained sections, defined as endothelial-lined luminal structures and the number of neovessels and the diameter of every neovessel exceeding 0.01 mm were recorded (10).

### Statistical methods

SPSS for Windows statistical software (version 29.0) was used for data analysis. Continuous variables were compared with Kruskal Wallis, and *post hoc* pairwise comparison tests were used to perform the statistical analysis. Coefficients of correlation were calculated by the Spearman ρ correlation for scale variables. The SMI count variable was treated as a continuous variable, including values of zero (i.e., no detectable intraplaque microvascular flow). All patients with available biomarker data were included in the correlation analyses, without exclusion based on SMI signal. The factors found significant were included in the linear multiple regression model. Due to the right-skewed distribution of sFlt-1 and sTie-2, a base-10 logarithmic transformation was applied to normalize the data and improve model fit. All statistical results were considered significant when *p* < 0.05.

## Results

Totally 44 patients were included, mean age was 72 ± 7 years and 28 (64%) were men. Twenty-nine had their blood drawn for the purpose of biomarker analysis, and 11 patients had their plaques removed at endarterectomy and assessed histologically under a microscope (**Table 1**; **Figure 1**). Among the 44 patients, 11 (25%) had no detectable intraplaque neovascularization (IPN) on SMI. In the remaining 33 patients with an IPN signal, the number of neovessels counted over a 2-minute SMI video ranged from 0 to 16; median 4 and mean 4.5. Plaque characteristics assessed by conventional ultrasound are summarized in **Table 2**.

**Table 1:** Patient characteristics.

Characteristic	Total cohort (N = 44)	Histology subgroup (N = 11)
Age (years)	71.7 ± 7.2	69.9 ± 6.7
Female sex, n (%)	16 (36.4)	4 (36.4)
Symptomatic, n (%)	30 (68.2)	9 (81.8)
Hypertension, n (%)	33 (75.0)	9 (81.8)
Diabetes mellitus, n (%)	6 (13.6)	1 (9.1)
Dyslipidemia, n (%)	21 (47.7)	4 (36.4)
Statin treatment, n (%)	39 (88.6)	10 (90.9)
Antihypertensive treatment, n (%)	28 (63.6)	7 (63.6)
Monotherapy/antiplatelet, n (%)	16 (36.4)	2 (18.2)
Dual antiplatelet therapy (DAPT), n (%)	27 (61.4)	9 (81.8)
Smoking (current/former), n (%)	26 (59.1)	5 (45.5)
BMI (kg/m²), N = 41	26.2 ± 3.9	25.7 ± 3.9
eGFR (mL/min/1.73 m²)	73.5 ± 16.3	71.0 ± 7.8
HbA1c (%), N = 42	5.46 ± 0.74	5.40 ± 1.7
Glucose (mmol/L), N = 43	6.14 ± 1.82	6.60 ± 2.51
Systolic BP (mmHg)	148.0 ± 26.7	143.9 ± 24.0
Diastolic BP (mmHg)	82.3 ± 20.7	80.0 ± 14.4
Creatinine (μmol/L)	83.6 ± 22.3	87.2 ± 14.8
Triglycerides (mmol/L), N = 41	2.39 ± 6.22	1.46 ± 0.60
HDL (mmol/L), N = 43	2.42 ± 1.16	1.16 ± 0.44
LDL (mmol/L), N = 43	2.31 ± 5.60	2.49 ± 0.95
Total cholesterol (mmol/L), N = 43	5.26 ± 7.52	4.07 ± 1.09
LDL/HDL ratio, N = 30	1.78 ± 1.14	2.40 ± 1.20
ESR (mm/hr)	10.6 ± 13.5	14.3 ± 13.3
Leukocytes (×10^9^/L)	10.4 ± 18.3	7.4 ± 1.47
CRP (mg/L)	4.56 ± 7.30	5.30 ± 9.70
IL-6 (pg/mL), N = 29	9.14 ± 29.67	19.76 ± 47.50
sFlt-1 (pg/mL), N = 27	71.9 ± 22.7	57.3 ± 13.8
Placental GF (pg/mL), N = 27	6.19 ± 1.98	5.94 ± 1.79
sTie-2 (pg/mL), N = 27	3849 ± 1013	3496 ± 657
VEGF-A (pg/mL), N = 27	58.2 ± 19.7	52.2 ± 19.5
VEGF-B (pg/mL), N = 27	13.5 ± 11.16	16.12 ± 13.11
VEGF-C (pg/mL), N = 27	19.9 ± 15.5	17.4 ± 14.5
VEGF-D (pg/mL), N = 27	933. ± 326.7	996.5 ± 377.5
VCAM-1 (ng/mL), N = 29	517 ± 116	567 ± 140
Angiopoetin-2, N = 27	774 ± 332	798 ± 423

LDL, Low-Density Lipoprotein cholesterol; HDL, High-Density Lipoprotein cholesterol; TG, Triglycerides; HbA1c, glycosylated Hemoglobin; eGFR, estimated Glomerular filtration rate; CRP, high-sensitivity C-reactive protein; ESR, sedimentation rate; VEGF, Vascular endothelial growth factor; VCAM-1, Vascular cell adhesion molecule.

**Table 2:** Plaque characteristics assessed by standard doppler ultrasound.

Plaque characteristics	Total cohort (N = 44)	Histology subgroup (N = 11)
Degree of stenosis (%)		
• 50–69% (ICA PSV 125–230 cm/s)	7 (15.9%)	2 (18.2%)
• ≥70% (ICA PSV >230 cm/s)	37 (84.1%)	9 (81.8%)
Plaque echogenicity		
• Hypoechoic	18 (40.9%)	5 (45.5%)
• Hyperechoic	26 (59.1%)	6 (54.5%)

### Relationship between neovascularization assessed by SMI, plaque histology, and growth factors in plasma

**Figure 3:**
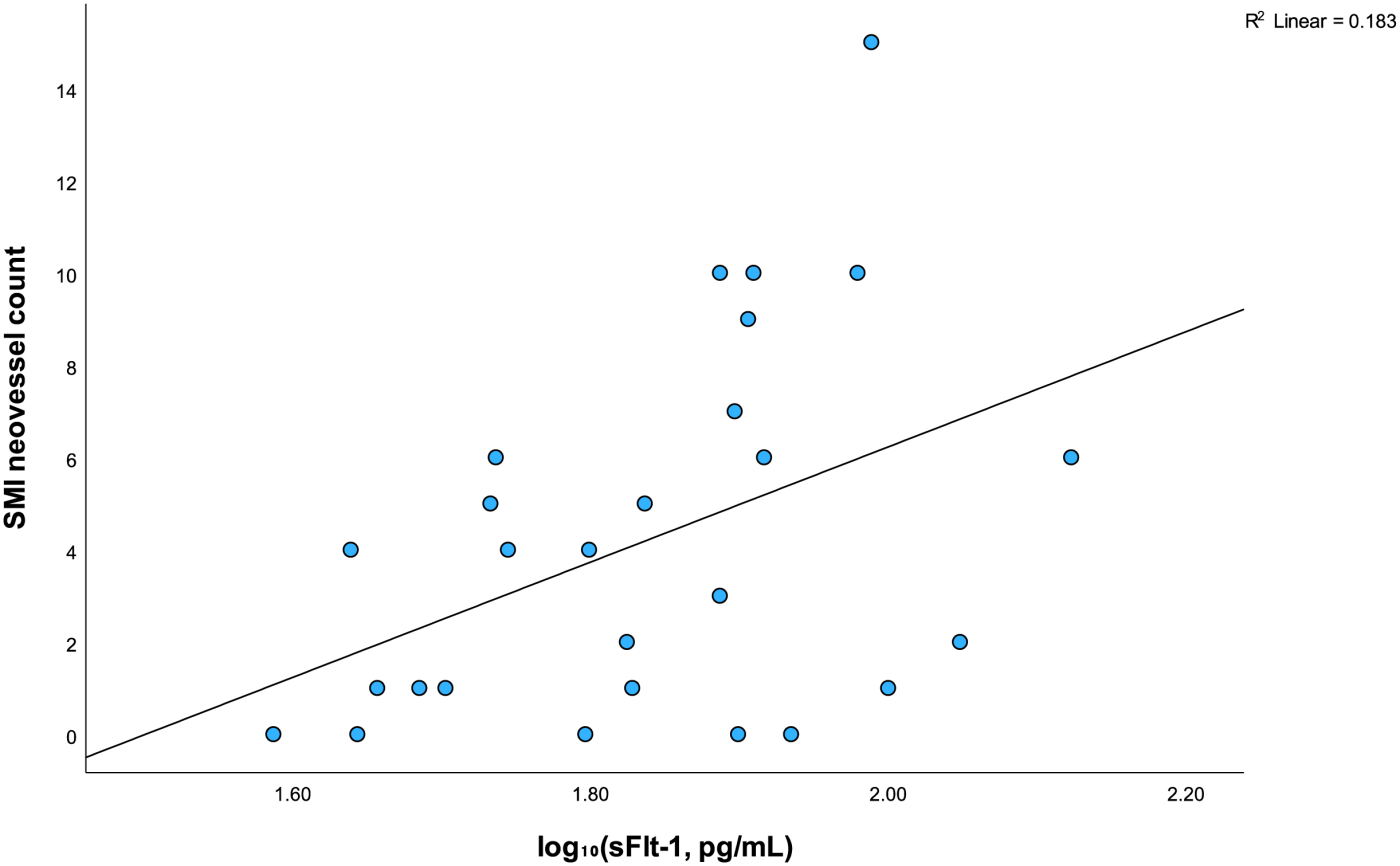
Scatter plot illustrating the relationship between plasma sFlt-1 levels (log_10_-transformed) and SMI neovessel count. A linear regression line is shown with the model explaining 18% of the variance (*R² = 0.18, p = 0.047*). The association remained significant after adjusting for BMI.

As shown in **Table 3** and **Figure 3**, plasma levels of sFlt-1 correlated significantly with SMI neovessel counts (*r=0.42*, *p=0.030, n=27*) with a similar trend for sTie-2 *(r=0.37, p=0.057, n=27)*. No such pattern was seen for VEGF-A/B/C/D or Ang-2 (**Table 3**). Moreover, in a subgroup of operated patients (n=11), the degree of neovascularization reflected by the number of counted neovessels as accessed by histological examination, was positively correlated with plasma sFlt-1 levels (*r=0.65, p=0.030)* and SMI neovessel count (*r= 0.68, p =0.02)*, but not with sTie-2 (**Table 3**).

**Table 3:** Correlation (Spearman) between plasma markers and SMI neovessel count (n=27, except histology subgroup n=11).

Biomarker/variable	ρ	P-value
sFlt-1 vs SMI	0.417	0.030
sTie-2 vs SMI	0.371	0.057
sFlt-1 vs Histology subgroup	0.651	0.030
SMI vs Histology subgroup	0.679	0.021
sTie-2 vs Histology subgroup	0.041	0.905
VEGF-A vs SMI	0.153	0.447
VEGF-B vs SMI	0.204	0.307
VEGF-C vs SMI (Spearman)	0.136	0.506
VEGF-D vs SMI	0.138	0.492
Angiopoietin-2 vs SMI	0.208	0.297

### Plasma levels of sFlt-1 and Tie-2 in relation to clinical and demographic characterization of the study group

Our findings so far suggest that sFlt-1 and to some degree also sTie-2 were related to the degree of IPN and were the focus of further analyses. As depicted in **Supplementary Table 1**, sFlt-1 (*r* = 0.46, *p* = 0.015), and sTie-2 (*r* = 0.47, *p* = 0.012), correlated with BMI and sTie-2 *(r=0.43, p=0.023)* also with age. Notably, sTie-1, but not sFlt-1, was significantly higher (*r*= −0.434, *p* = 0.024) in symptomatic versus asymptomatic patients (**Supplementary Table 1**).

In contrast, no significant associations were found with other cardiovascular risk factors such as lipid profile, inflammatory blood parameters including CRP and leukocyte counts *and* SMI neovessel assessment, *nor* plasma sFlt-1 and sTie-2 levels (**Supplementary Tables 1**, **S2**).

### sFlt-1 is independently associated with SMI-assessed neovessel counts

Since age and adipose tissue affect vascular biology, including endothelial dysfunction and angiogenesis (21), we assessed whether age and BMI would influence the association between sFlt-1 and intraplaque neovascularization. However, linear regression analysis revealed that sFlt-1 was significantly associated with higher SMI assessed neovessel counts *(B = 13.24, 95% CI: (0.17, 26.30), p=0.047)*, independent of age and BMI. The model explained approximately 18% of the variance in neovessel count. In contrast, log-transformed Tie-2 levels were not significantly associated with SMI neovessel count in models adjusted for BMI or age (**Table 4**).

**Table 4:** Linear regression models for SMI neovessel count with log-transformed plasma markers.

Model	B (log-marker)	95% CI	p-value	Adjusted for	R²
SMI ~ log_sFlt-1 + BMI	13.24	[0.17, 26.30]	0.047	BMI	0.184
SMI ~ log_sTie-2 + BMI	–1.51	[–11.89, 8.88]	0.767	BMI	—

### Cell distribution of Flt-1, VEGF-A, TEK, Ang-1 and Ang-2 RNA expression

**Figure 4:**
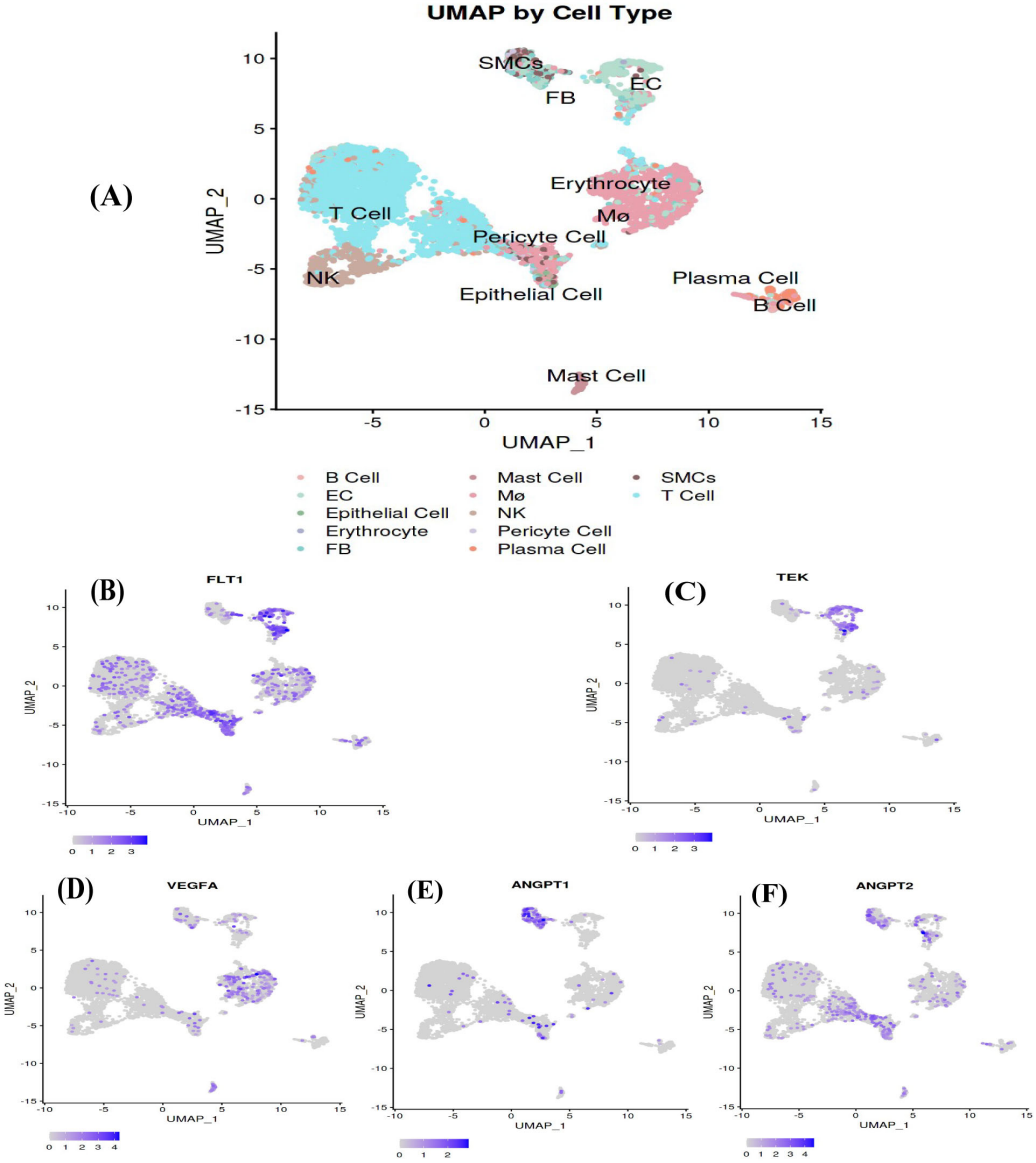
Analysis of publicly available Single‐cell transcriptome data from carotid plaque by Slenders et al. (PMID: 35174364) from 38 patients with carotid atherosclerosis **(A)** Seurat Clusters labelling for cell identification, Expression levels across the different clusters of FLT1 **(B)**, TEK **(C)**, VEGF-A **(D)**, ANGPT1 **(E)**, ANGPT2 **(F)**.

To investigate if Flt-1, VEGF-A, TEK, Ang-1 and Ang-2 could be synthesized within the carotid plaques. We examined the RNA expression levels of these proteins in relation to cell types in carotid plaques by accessing publicly available single‐cell transcriptome data from carotid plaques by Slenders et al. (PMID: 35174364) from 38 patients with carotid atherosclerosis (18). Analysis of these available data files showed that Flt-1 and Ang-2 had a general wide vascular distribution amongst the different clusters, while TEK is mainly expressed in the Endothelial cell cluster, Ang-1 mainly in the smooth muscle cell (SMC) cluster and VEGF-A is mainly expressed in the erythrocytes cluster (**Figure 4**).

## Discussion

We investigated the relationship between intraplaque neovascularization (IPN) and circulating levels of the angiogenic regulators sFlt−1 and sTie−2 in patients with carotid atherosclerosis. Our findings demonstrate that IPN is associated with higher circulating levels of angiogenic regulators, particularly sFlt−1 and, to a lesser extent, sTie−2.

In both imaging and histological analyses, plaques with more extensive neovascularization had higher circulating sFlt−1 levels, and for sFlt−1 this relationship remained significant after adjustment for age and BMI, suggesting that the association with IPN is not simply a reflection of age related changes or adiposity−related sFlt−1 variation (22, 23).

sFlt−1 acts as a decoy receptor and attenuates VEGF−mediated angiogenic signaling (24). Experimental models show that it´s overexpression reduces neointimal formation and plaque microvessel density, supporting an anti−angiogenic, plaque−stabilizing role in the arterial wall (25, 26). In humans, increased circulating sFlt−1 is best known from preeclampsia and peripartum cardiomyopathy, where placental hypoxia induces sFlt−1 overproduction with accompanying systemic endothelial dysfunction (27, 28). IPN are thought to arise in response to plaque growth, hypoxia, and inflammatory activation, but they are immature and leaky and contribute to plaque vulnerability. Our observation that higher sFlt−1 levels are associated with higher IPN may therefore reflect an endogenous anti−angiogenic feedback to limit further VEGF−driven neovessel formation in an already diseased vascular bed. However, this interpretation is speculative and should be viewed as one of several possible explanations for the association between sFlt−1 and IPN in this small, cross−sectional cohort. However, whether sFlt−1 overproduction represents primarily a hypoxia−driven response occurring in parallel with IPN, or a more targeted negative−feedback mechanism to counteract intraplaque angiogenesis, remains uncertain and warrants confirmation in larger cohorts.

Alternative, non−mutually exclusive interpretations are also plausible. First, circulating sFlt−1 has been linked to endothelial dysfunction in other vascular beds, correlating independently with von Willebrand factor and soluble VCAM−1 and experimentally inducing endothelial apoptosis, reduced nitric oxide bioavailability, and impaired angiogenesis (29, 30), suggesting that elevated sFlt−1 may primarily reflect systemic or plaque−level endothelial injury rather than a targeted feedback loop. Second, experimental work has shown that sFlt−1 antagonizes autocrine VEGF−A signaling and thereby renders endothelial cells more sensitive to pro−inflammatory cytokines such as TNF−α, which amplifies cytokine−driven endothelial activation and leukocyte adhesion (31) instead of initiating activation *de novo*. In the context of atherosclerotic plaques, where inflammatory cytokines are abundant, higher sFlt−1 could thus mark a state of heightened endothelial susceptibility to inflammatory stress within neovessel−rich, vulnerable plaques.

To explore the relevance of these angiogenic regulators within the plaque, we interrogated publicly available single−cell RNA−seq data from human carotid endarterectomy specimens (18). In keeping with their canonical endothelial roles, TEK (Tie−2) expression was largely restricted to endothelial cell clusters, whereas Flt1 and Ang-2 showed broader expression across endothelial cells, smooth muscle cells, and myeloid subsets. this cellular distribution confirms, in human carotid endarterectomy tissue, that VEGF–FLT1 and Ang–TEK/Tie−2 pathways are present in endothelial, smooth−muscle and myeloid compartments where IPN and inflammation develop. These exploratory data are descriptive and do not establish a causal link between circulating sFlt−1/sTie−2 and plaque behavior, but they support the biological plausibility that systemic alterations in these pathways may reflect signaling activity within the plaque microenvironment (32).

Clinically, the diverse roles of sFlt−1 and Tie−2 are reflected across different vascular beds. Low sFlt−1 has been implicated in conditions characterized by pathological ocular neovascularization, such as diabetic retinopathy and neovascular age−related macular degeneration (33, 34), whereas higher sFlt−1 levels appear protective in these settings by restraining excess angiogenesis. Findings from the large Dallas Heart Study provide further support for a broader vascular relevance of sFlt−1. In a population−based sample, higher sFlt−1 levels were independently associated with subclinical aortic atherosclerosis and predicted future atherosclerotic cardiovascular events, even after adjustment for conventional risk factors (15). Taken together, our data suggest that elevated sFlt−1 in patients with marked IPN may reflect a partially effective anti-angiogenic feedback to VEGF-driven microvascular stress that may coincide with systemic endothelial dysfunction.

Similarly, altered Ang/Tie−2 signaling has been linked to atherosclerotic disease, and experimental Ang−2 blockade or Tie−2 preservation can reduce plaque progression and vascular leakage, suggesting that this pathway modulates both vascular stability and inflammation (35, 36). In contrast to sFlt-1, associations between sTie-2 and intraplaque neovascularization were weak and inconsistent in this pilot cohort, despite a significant correlation between SMI-derived neovessel count and histological neovessel count in the subgroup with both assessments (*r= 0.68, p =0.021*). Because soluble Tie-2 is generated by shedding of the endothelial Tie-2 receptor and can bind both Ang-1 and Ang-2, it is thought to modulate the overall balance of Ang/Tie-2 signaling rather than serving as a specific marker of either vessel stabilization or destabilization (14, 37, 38), and elevated sTie-2 has been linked to endothelial activation, vascular leakage and inflammation (39, 40). Circulating sTie-2 may therefore primarily reflect dysregulation of the Ang/Tie-2 axis and endothelial barrier function during active angiogenic remodeling, rather than being tightly proportional to the number of relatively larger, structurally established intraplaque microvessels quantified histologically. In our study, histological neovascularization was assessed on H&E sections and restricted to intraplaque vessels with a diameter ≥0.01 mm, which favors larger microvessels and may underestimate smaller or immature neovessels. Together with the limited size of the histology subgroup, these factors may explain the absence of a significant association between sTie-2 and histological neovessel density despite a trend with SMI, and we regard the sTie-2 findings as inconclusive but not inconsistent with a potential role of the Angiopoietin/Tie-2 axis in plaque vulnerability (35, 41).

The principal limitation of our study is a low sample size and the cross-sectional design, underscoring that our data need to be confirmed in larger cohorts that also include longitudinal follow-up with outcome data. The fact that not all examinations were performed in all patients is also a limitation of the study. Another limitation of this study is that histological assessment of neovascularization was based on hematoxylin and eosin staining without immunohistochemical characterization of endothelial or pericyte markers. Therefore, we could not directly assess neovessel maturity or permeability. Future studies incorporating specific vascular markers (e.g., CD31, α-SMA) are needed to further characterize vessel phenotype and its relationship to circulating angiogenic factors. Forthcoming studies should also include systematic assessment of fibrous cap thickness, a key marker of plaque stability, to relate circulating sFlt−1 levels and SMI−defined IPN more directly to cap vulnerability.

Moreover, associations do not necessarily mean any causal relationship and in the present study, several comparisons were performed and some of them could be by chance. Several variables could have influenced our data such as sex, inflammation and diabetes but due to the limited total sample size the number of co-variants that could be included were restricted to age and BMI. The strengths are the well-clinically defined group, including the advanced and accurate carotid ultrasound examinations as well as histological assessments.

In conclusion, in this pilot study, higher plasma sFlt−1, and to a lesser extent sTie−2, were associated with carotid IPN on SMI and for sFlt-1, by histology, suggesting that circulating components of the VEGF and Ang/Tie−2 pathways may function as candidate, context−sensitive markers of intraplaque angiogenesis. However, effect sizes were modest, associations for sTie−2 were not statistically robust, and our cross−sectional design without outcome data does not allow firm conclusions about a mechanistic link to plaque vulnerability. Accordingly, our results should be viewed as hypothesis−generating and require confirmation and extension in larger, longitudinal cohorts with clinical endpoints and to clarify the temporal relationship between these biomarkers and IPN, and ultimately plaque vulnerability.

## Data Availability

The raw data supporting the conclusions of this article will be made available by the authors, without undue reservation.
