# Supplementary material for: Plasma levels of soluble Flt-1 and Tie-2 correlate with neovascularization in human carotid atherosclerotic plaques – a pilot study
Source: Front Immunol. 2026 Jul 21;17:1791571. doi: 10.3389/fimmu.2026.1791571 (PMC13433193; doi:10.3389/fimmu.2026.1791571)
Supplement: Supplementary file 1 [file Table1.docx]

## Supplemental Table 1: sFlt-1 and sTie-2 Correlations with Clinical and Laboratory Variables (N=27)

|  | sFlt-1 | | sTie-2 | |
| --- | --- | --- | --- | --- |
| Variables | ρ | p-value | ρ | p-value |
| Age | 0.235 | 0.238 | 0.437 | 0.023 |
| Gender | 0.079 | 0.696 | -0.098 | 0.625 |
| Smoking | 0.077 | 0.704 | -0.038 | 0.850 |
| Diabetes mellius | -0.04 | 0.842 | -0.187 | 0.349 |
| Hypertension | -0.013 | 0.947 | -0.388 | 0.045 |
| Hypercholesterolemia | -0.048 | 0.814 | -0.447 | 0.019 |
| BMI | 0.462 | 0.015 | 0.477 | 0.012 |
| Symptoms | 0 | 1 | –0.434 | 0.024 |
| Statin medication | 0.106 | 0.599 | -0.076 | 0.708 |
| Aspirin | -0.049 | 0.808 | -0.086 | 0.671 |
| CRP | -0.049 | 0.807 | -0.476 | 0.012 |
| Leukocytes | 0.276 | 0.164 | -0.089 | 0.659 |
| Cholesterol | 0.011 | 0.955 | 0.162 | 0.420 |
| HDL | 0.119 | 0.556 | 0.127 | 0.528 |
| LDL | -0.118 | 0.559 | -0.039 | 0.846 |
| TG | 0.079 | 0.696 | 0.234 | 0.239 |
| Glucose | -0.044 | 0.828 | 0.045 | 0.823 |
