# Supplementary material for: Plasma levels of soluble Flt-1 and Tie-2 correlate with neovascularization in human carotid atherosclerotic plaques – a pilot study
Source: Front Immunol. 2026 Jul 21;17:1791571. doi: 10.3389/fimmu.2026.1791571 (PMC13433193; doi:10.3389/fimmu.2026.1791571)
Supplement: Supplementary file 2 [file Table2.docx]

## Supplemental Table 2: SMI Count Correlations with Clinical and Laboratory Variables (n=29)

| **Biomarker / Variable** | **ρ** | **p-value** |
| --- | --- | --- |
| SMI vs Age | 0.349 | 0.064 |
| SMI vs Gender | -0.193 | 0.316 |
| SMI vs Smoking | -0.253 | 0.185 |
| SMI vs Diabetes mellitus | -0.145 | 0.454 |
| SMI vs Hypertension | -0.066 | 0.734 |
| SMI vs Hypercholesterolemia | -0.242 | 0.205 |
| SMI vs BMI | 0.199 | 0.309 |
| SMI vs Statin medication | 0.218 | 0.255 |
| SMI vs Aspirin | -0.164 | 0.395 |
| SMI vs CRP | -0.34 | 0.071 |
| SMI vs Leukocytes | 0.024 | 0.902 |
| SMI vs Cholesterol | 0.115 | 0.553 |
| SMI vs HDL | 0.172 | 0.447 |
| SMI vs LDL | -0.103 | 0.596 |
| SMI vs TG | 0.19 | 0.323 |
| SMI vs Glucose | 0.182 | 0.346 |
